# Spindle Positioning, Meiotic Nonreduction, and Polyploidy in Plants

**DOI:** 10.1371/journal.pgen.1000272

**Published:** 2008-11-28

**Authors:** Sebastien Andreuzza, Imran Siddiqi

**Affiliations:** Centre for Cellular and Molecular Biology, Hyderabad, India; The University of North Carolina at Chapel Hill, United States of America

Polyploidy, the state of having more than two sets of chromosomes, is common in flowering plants (angiosperms), including the major crops [1]. Indeed, it is estimated that 30%–80% of the angiosperms are polyploids [2], and most diploid plant species, including *Arabidopsis thaliana*, show evidence of genome duplication in their ancestry [3]. Polyploidy is accompanied by genome-wide changes in gene expression and epigenetic modifications, leading to phenotypic diversity and rapid adaptation [4]. Doubling of chromosome number can also rescue and stabilize interspecific hybrids that would otherwise show a high degree of sterility due to failures in meiosis. Thus, polyploidy is thought to be a driving force in plant evolution and speciation [5]. In this issue of *PLoS Genetics*, d'Erfurth and colleagues report on the molecular characterization of the *AtPS1* gene and show that lesions in the gene result in unreduced gametes, which are believed to be an important step in polyploidization [6].

While polyploids can originate by an increase of chromosome number either during somatic growth or during meiosis, the major route is now considered to be via the formation of unreduced gametes [7]. The formation of 2n gametes resulting from failure of reduction during meiosis occurs in several plant species and can give rise to triploids that can serve as a bridge to the establishment of an even set of chromosomes in subsequent generations [8]. Unreduced gametes have also been used in breeding schemes for crop improvement wherein beneficial traits have been introduced from diploid relatives into cultivated polyploid species [9].

**Figure 1:**
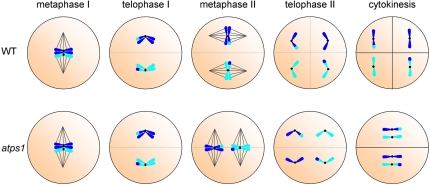
Formation of parallel spindles during meiosis II in *atps1* gives unreduced spores. In *atps1* formation of meiosis II, spindles in a plane parallel to that during meiosis I results in each half of the cell getting one of the two sister chromatids. Cytokinesis occurs only at the end of meiosis II, and the planes of division overlap, resulting in a dyad of diploid spores.

The paper by d'Erfurth et al. [6] provides the first molecular insight into high-efficiency unreduced pollen formation in plants based on analysis of mutants in the *Arabidopsis* gene *AtPS1* that produce diploid pollen. A range of meiotic abnormalities can give rise to unreduced gamete formation. These can be broadly classified as first or second division restitution (FDR and SDR, respectively) [2]. The occurrence of parallel spindles at meiosis II, resulting in a single plane of cell division and formation of dyad spores, is a frequently found mechanism for 2n gamete formation and is genetically equivalent to FDR in that it results in retention of heterozygosity for centromeric markers (Figure 1). The *parallel spindle 1* (*ps1*) mutant of potato, which produces unreduced pollen by such a mechanism, was described more than 80 years ago [10] and has been used in potato breeding for introgression of beneficial traits in cultivated strains [11]. While the occurrence of unreduced gamete formation in plants has long been recognized, the molecular mechanisms leading to diploid gamete formation have only recently begun to be uncovered [12],[13].

d'Erfurth et al. identified the *AtPS1* gene using a novel bioinformatics approach. They looked for genes whose expression pattern in microarrays correlated with that of known meiotic genes. Subsequent analysis showed that T-DNA insertions in *AtPS1* result in the production of diploid pollen, and triploidy in as many as 30% of progeny. The authors then show, by elegant genetic and cytological experiments, that the *atps1* mutant phenotype results from the positioning of the spindles at metaphase II in a parallel orientation instead of in the normal tetrahedral configuration. In most angiosperms, including *Arabidopsis*, cytokinesis at the time of meiosis occurs only after the second round of chromosome segregation. The occurrence of parallel spindles at meiosis II in *atps1* thus results in the formation of one plane of division, and gives rise to dyads containing diploid spores instead of a tetrad of haploid spores (Figure 1).

Interestingly, *AtPS1* is a plant-specific gene that encodes a protein containing an N-terminal Forkhead Associated (FHA) domain. FHA domains are phosphoprotein binding modules and are found in proteins that regulate signal transduction pathways in several different contexts: plant meristem homeostasis, DNA repair, and cell cycle control [14]. The identification of *AtPS1* opens a new window on the control of meiosis II, particularly in relation to spindle orientation.

The spindle pole body (SPB) and the centrosome are the yeast and animal microtubule organizing centers (MTOCs), respectively. MTOCs nucleate microtubules and orient the spindle during cell division [15]. Within the SPB and centrosome is a pair of centrioles that contain γ-tubulin, the site of nucleation of microtubules [16]. Although γ-tubulin is conserved in eukaryotes, land plants lack a conspicuous MTOC in the form of a centrosome. Nucleation of microtubules occurs in the cortical cytoplasm and on the nuclear envelope from sites along existing microtubules that are marked by γ-tubulin [17]. Microtubule nucleation in plants therefore appears to be spatially distributed within the cell. A number of reports point to γ-tubulin as the plant MTOC, but the precise mechanism underlying orientation of the spindle and its relation to spindle biogenesis during cell division in plants remains to be established [18]. The identification of *AtPS1* and its role in controlling spindle orientation during meiosis II represents an important step in understanding the control of spindle positioning in plants, and determination of its mode of action would be of continued interest. Importantly, because unreduced gametes are of agronomic value, the discovery of *AtPS1* may also benefit crop biotechnology.
